# Effects of “Living High‐Training Low and High” on Sleep, Heart Rate Variability, and Psychological Responses in Elite Female Cyclists

**DOI:** 10.1002/ejsc.12320

**Published:** 2025-07-15

**Authors:** Thibaud Pirlot, Thibaud Mihailovic, Philippe Gimenez, Gregoire P. Millet, Franck Brocherie, Eric Fruchart, Gilles Ravier, Bertrand Baron, Romain Bouzigon, Sandrine Guirronnet, Emmanuel Brunet, Alain Groslambert

**Affiliations:** ^1^ Laboratory C3S, University Marie & Louis Pasteur Besançon France; ^2^ Labcom C3S/Équipe Cycliste Groupama‐FDJ Besançon France; ^3^ ITA 3.0 Besançon France; ^4^ ISSUL Quartier UNIL‐Centre Lausanne Switzerland; ^5^ Laboratory Sport Expertise and Performance (EA 7370) French Institute of Sport (INSEP) Paris France; ^6^ UR 4604 UPVD Font Romeu France; ^7^ Espace‐Dev Montpellier Montpellier France; ^8^ FFC Montigny‐le‐Bretonneux France

**Keywords:** acclimatization, altitude training, perceived exertion, performance, recovery, stress

## Abstract

“Living High‐Training Low and High” (LHTLH) is an altitude/hypoxic training method used to improve physical performance at sea level. The aim of this exploratory study was to investigate the effects of LHTLH on sleep, heart rate variability (HRV), and psychological stress in 10 elite/international level female cyclists (mean age: 17.3 ± 1.2 years). Participants were monitored for 19 days divided into 3 periods: (i) normoxia (5 days preceding LHTLH), (ii) early acclimatization (day 1–4 of LHTLH), and (iii) middle acclimatization (day 5–14) performed in hypoxic rooms (F_i_O_2_ = 15.09%). Questionnaires of psychological stress and sleep disturbance, sleep architecture (determined through an electroencephalography sleep headband), and HRV (measured at rest with a chest strap) were recorded during the 3 periods. The results found that, compared to normoxia, there was no significant difference in total sleep time, sleep efficiency, latency, or waking during the early acclimatization period. However, a significant increase in sleep disturbance (2.5 ± 1.1 vs. 4.9 ± 2.5 a.u. and *p* < 0.001), alterations of HRV, and sleep architecture with a significant increase in stages 1 (21.8 ± 3 vs. 25.9 ± 3.6 min and *p* < 0.007) and 2 (201.2 ± 55 vs. 238.5 ± 55 min and *p* < 0.008) of sleep was observed. During the middle acclimatization period, the athletes had restorative sleep but HRV remained altered, with a significant increase in external tension (1.24 ± 0.4 vs. 2.83 ± 1.8 a.u. and *p* < 0.05). All these findings suggest that an acclimatization period of at least 4 days is required to recover restorative sleep during LHTLH intervention.

– This article provides the first insights on the understanding of psychophysiological stress induced using the “Live High‐Training Low and High” paradigm, particularly in elite/international level female athletes.
– Results indicate that:
  - during the early acclimatization period, sleep disturbances are observed, with an alteration in heart rate variability.
  - After this period, the athletes return to restful sleep, enabling them to increase their training load.
  - However, heart rate variability and psychological stress remain impaired for at least 10 days.

**Summary**

- Sleep quality was impaired during the first 4 nights of LHTLH exposure in elite female cyclists.
- Sleep progressively normalized after 4 days, but heart rate variability and psychological stress remained altered.
- A minimum of 4 days of acclimatization is recommended before increasing the training load in hypoxia.
- Continuous monitoring of sleep, HRV, and psychological stress is crucial during LHTLH interventions.

## 1 Introduction

“Living High‐Training Low and High” (LHTLH) is an altitude/hypoxic training method used by athletes to improve both aerobic and anaerobic performance at sea level (Brocherie et al. 2015; Millet et al. 2010). The main concept is based on the combination of chronic “Living High‐Training Low” altitude residence that stimulates hematological adaptations (mainly through hemoglobin mass increase), with additional “Repeated‐Sprint training in Hypoxia” (RSH; Brocherie et al. 2017) which, through the repetition of short (< 30 s) “all‐out” sprints with incomplete recoveries, induces muscle and microvascular adaptations leading to higher resistance to fatigue (Brocherie et al. 2015, 2018). Indeed, since LHTLH is a novel hybrid altitude/hypoxic strategy, it is paramount to investigate its effects on sleep, heart rate variability (HRV), and psychological stress. In addition, although RSH has been developed and adopted since 2013 for team‐sports athletes (Millet et al. 2013; Faiss et al. 2013), it was embraced very early in many endurance sports (Faiss et al. 2015). Due to the intermittent pacing observed in many endurance sports (i.e., cross‐country skiing or cycling), delaying muscle fatigue to repeated high‐intensity short exercise bouts appears relevant. Previous reports confirm that RSH is performed by many professional cyclists (e.g., Faiss and Rapillard 2020). Given the relevance of the LHTLH approach, it appears interesting to further investigate in situ LHTLH intervention in elite/international cyclists.

Although recognized as a relevant strategy for improving endurance performance both at altitude and sea‐level (Millet and Brocherie 2020; Millet et al. 2019), altitude/hypoxic training methods require a careful scheduling of the hypoxic exposure and an adjustment of training content and training loads (TLs) (Chapman 2013; Constantini et al. 2017). Moreover, acute and/or chronic hypoxic exposure is also known to alter sleep (G. D. Roach et al. 2013) notably with reduced total sleep time, lower sleep efficiency, and modification of sleep architecture (Hoshikawa et al. 2007; Hrozanova et al. 2021) and a decrease in rapid eye movement (REM) and/or non‐REM (NREM) stages (Heinzer et al. 2016). Mechanisms underlying these changes involve altered chemoreceptor sensitivity and hypoxic ventilatory response, which contribute to frequent arousals and fragmented sleep (Weil 2004). Because endurance athletes sleeping in hypoxia could present impaired recovery, mainly due to periodic breathing, increased frequency of hypopneas and desaturation events (i.e., oxygen desaturation index [ODI] = 3%) compared to normoxia (Heinzer et al. 2016; Saugy et al. 2016), understanding the association between altitude training and sleeping pattern would be an asset.

Hypoxia is also an environmental condition that affects cardiac autonomic responses, which can be assessed by HRV, defined as the fluctuations in R‐wave to R‐wave intervals (Oliveira et al. 2017). In acute hypoxia, it is generally accepted that short‐term exposure to hypoxia results in decreased resting HRV and sympathetic dominance and withdrawal of vagal control (Schmitt et al. 2008). After acclimatization, the athletes generally increase the LF component and LF/HF ratio during exercise through the activation of the sympathetic system (Povea et al. 2005; Schmitt, Regnard, et al. 2018). Moreover, it has been reported that HRV during postexercise recovery is not only modulated by the characteristics (intensity and duration) of the previous exercise (Povea et al. 2005) but also by intense emotions (Wei et al. 2017).

In addition to physiological effects, hypoxia impacts psychological stress and emotions. For instance, an increased anger has been reported as well as an increase in perceived exertion, which all tend to impair the athlete's performance (Jeffries et al. 2019). Although some studies report elevated tension and confusion (Keramidas et al. 2016), others describe improved mood and positive affective responses (Seo et al. 2015). Thus, some moderator variables, such as personality traits, could explain these individual differences (McMorris 2020). For this reason, it appears interesting to investigate the effects of LHTLH on the hierarchical dispositional trait composed of two major factors involved in high‐level sports, namely, perseverance of effort and consistency of interest. Perseverance of effort involves diligently overcoming adversity in pursuit of challenging goals, whereas consistency of interest emphasizes the focus on goals over time (Duckworth and Quinn 2009). A major challenge for coaches is to know whether chronic hypoxic exposure, such as LHTLH, influences psychological stress, emotions, and dispositional traits, which could in turn affect the engagement of athletes during training.

Although altitude/hypoxia's effects on sleep, HRV, and psychological stress have been widely studied in “Living High‐Training High” or LHTL interventions (Millet et al. 2010; Heinzer et al. 2016), research on LHTLH remains scarce. Furthermore, most studies have focused on male or mixed‐gender groups, with limited investigation of female athletes (Schmitt, Willis, et al. 2018). Therefore, the aim of this exploratory study was to investigate the influence of LHTLH on sleep, HRV, and psychological stress, including emotions and personality, in 10 elite/international level female track endurance cyclists. We tested the hypothesis that compared to normoxia, sleep, HRV, and psychological stress would be impaired using LHTLH but return to normal after an early acclimatization phase.

## 2 Methods

### 2.1 Participants

Ten Caucasian female track endurance cyclists with an elite/international level (U19 and U23 national team; Tier 4) according to McKay et al. (2022) volunteered to participate to the study. Menstrual status was determined with the counting days method and the estradiol and progesterone dosage (Carmichael et al. 2021) and revealed 4 women in the follicular phase, 3 women in the luteal phase, 1 woman in the ovulation phase, and 2 women with amenorrhea at the first day of LHTLH. All the participants used oral contraception. Six weeks before intervention, a blood sample was taken to control iron status and all participants were prescribed an iron supplement (80 mg of Tardyferon per day during the 4 weeks preceding the LHTLH camp and one dose of Vitamin D Uvedose). During LHTLH, urine specific gravity (density) was measured daily with a refractometer (Pen‐urine SG 3741, ATAGO Co Ldt Japan) to verify the participant's hydration status. The morningness–eveningness in circadian rhythms (Horne and Östberg 1976) was self‐assessed 1 week prior to LHTLH. To avoid disrupting nighttime sleep measurements (Rea et al. 2022), participants were instructed to refrain from taking naps during the protocol. After consultation, none of them reported using naps as a habitual practice. In addition, participants were instructed to refrain from consuming caffeine throughout the entire protocol. The participants or their legal representative gave their written informed consent for participation in the investigation. The experiments complied with a national sport ethic committee (#ReFORM) and the Helsinki Declaration (1975 and revised in 2013).

The main characteristics of the participants are presented in Table 1.

**TABLE 1:** Main characteristics of the participants.

Physical and behavioral characteristics	
Participants	10 women
Cycling level	National to international
Discipline	Road and track endurance
Mean age (years)	17.3 ± 1.2
Body mass index (kg.m^2^)	22.5 ± 2.1
VO_2_ max (mL.min.kg^−1^)	54.9 ± 6.9
Nap	None
Chronotype of sleep	Mostly not eveningness or morningness

### 2.2 Procedure

**FIGURE 1:**
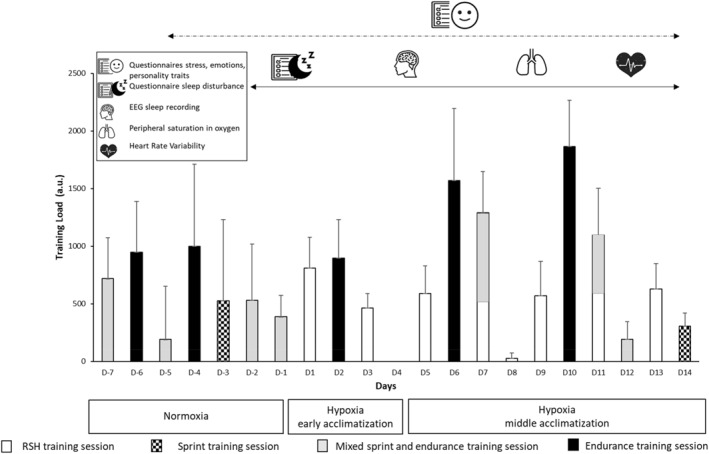
Experimental design of the study. The bars correspond to the training load. EEG = electroencephalography; RSH = repeated‐sprint training in hypoxia.

Figure 1 presents the protocol, which was designed as an explanatory within‐subject repeated measure. Participants were monitored for 19 days divided into 3 periods: (i) normoxia (5 days preceding LHTLH; at altitude between 130 and 500 m), (ii) early acclimatization (Day 1–4) of LHTLH; at simulated altitude of 2800 m (fraction of inspired oxygen [F_i_O_2_] = 15.09%), to prevent acute mountain sickness (AMS) and fatigue (Clark and Sheraton 2023), and (iii) middle acclimatization (Day 5–14) at simulated altitude of 2800 m (F_i_O_2_ = 15.09%), with a progressive increase in TL.

#### 2.2.1 Normoxic Period

For 5 days, the participants trained (road and track cycling) and slept in their usual training center and completed 3 questionnaires on stress, emotions, and personality every other day. Also, during the last 2 nights in normoxia, baseline measurement was performed for sleep disturbance, AMS, sleep architecture, peripheral oxygen saturation (SpO_2_, in %), continuous nocturnal heart rate (HR, in bpm), and HRV. On the last day of the normoxic period, maximal oxygen consumption (VO_2_max, in mL.min^−1^.kg^−1^) was measured with a maximal aerobic power test for characterization of participants.

#### 2.2.2 Early Acclimatization (Day 1–4) of LHTLH

During LHTLH, the participants spent ∼14 h per day in the training center's hypoxic dormitories set at a simulated altitude of 2800 m for sleep and posttraining recovery. The reduction in F_i_O_2_ was maintained constant (F_i_O_2_ = 15.09%, barometric pressure = 730 mmHg; inspired partial pressure of oxygen [P_i_O_2_] = 103 mmHg) using an oxygen extraction system (Altitude generator, type 85, B‐Cat, Netherlands), which was calibrated at the beginning of the study, with the level of oxygen and carbon dioxide continuously controlled. Two RSH sessions (days 1 and 3) performed in the laboratory in controlled environmental conditions (20°C, 60% humidity) at a simulated altitude of 3000 m (F_i_O_2_ = 14.68%; barometric pressure = 730 mmHg; and P_i_O_2_ = 103 mmHg) were included during the early acclimatization period. RSH consisted of a standardized 10 min warm‐up (resistance: 0.2 Nm.kg^−1^), then followed by 3 sets of 5 “all‐out” sprints of 10 s (resistance: 0.6 Nm. kg^−1^) interspersed by 20 s of passive recovery (resistance: 0.0 Nm.kg^−1^) with 5 min of active recovery (resistance: 0.2 Nm.kg^−1^) between the sets. RSH was carried out on a magnetic resistance bicycle fitted with an SRM sensor (Schoberer Rad Messtechnik, Welldorf, Germany). The LHTLH “training‐low” phase was performed between 300 and 500 m on day 2 and consisted of outdoor endurance cycling training around the training center's hypoxic dormitories. Day 4 was designated as a recovery day.

#### 2.2.3 Middle Acclimatization (Day 5–14) of LHTLH

The participants continued the LHTLH training camp in the same hypoxic conditions as those during the early acclimatization period, with the inclusion of five RSH sessions on days 5, 7, 9, 11, and 13. The LHTLH “training‐low” phase was performed between 300 and 500 m (days 6, 7, 8, 10, 11, 12, and 14) and consisted of sport‐specific outdoor training, including endurance, sprint, and mixed sprint, and endurance training sessions around the training center's hypoxic dormitories.

All the measurements performed during the normoxic period every day (i.e., sleep architecture, sleep disturbance, HRV, and SpO_2_) and every other day (i.e., questionnaires) were reproduced during the early and middle acclimatization periods. The normoxic‐related and LHTLH‐related trainings were determined with the head coach and were composed of endurance and/or sprint cycling training sessions performed in normoxia or hypoxia (Figure 1). The TL was daily recorded during all the experiment. To avoid possible tiredness, the questionnaires on stress, emotions, and personality were completed every other day. Therefore, participants were asked to average their psychological responses over the two last days.

### 2.3 Material

Sleep architecture was recorded using an electroencephalography sleep headband (Dreem; https://dreem.com, Dreem, Science Team, New York, NY). The validity of this device has been previously reported in reference to polysomnography for electroencephalographic signal acquisition and sleep staging (Arnal et al. 2020). Participants were equipped with the device and sleep architecture, including stages 1, 2, and 3, of the nonrapid eye movement (NREM1, NREM2, and NREM3, respectively) and stage of the rapid eye movement (REM) was recorded during sleep. NREM1 and NREM2 are considered as light sleep, whereas NREM3 corresponds to deep sleep and REM represents a phase in which the individual dreams vividly. The following standard sleep variables were investigated with the sleep headband: total sleep time (TST, in min); sleep efficiency (ES, in %), as TST/TIB × 100, where time in bed (TIB, in min) is the duration from lights‐out to lights‐on; sleep onset latency (SOL, in min) is the duration from lights‐out to the first three consecutive epochs of any sleep stage; wakefulness after sleep onset (WASO, in min), as the duration awake following the first three consecutive epochs of any sleep stage; and the time (in min) spent in each sleep stage (NREM1, NREM2, NREM3, and REM).

HRV was recorded with a chest strap HR monitor that records R‐wave to R‐wave intervals (Polar V800, Kempele, Finland). The R‐wave to R‐wave interval samples were recorded with a sampling rate of 1000 Hz with a HR monitor. The data recorded were downloaded via the Polar FlowSync software and exported for analysis by using the Kubios HRV software v3.0.0 2 (Biosignal Analysis and Medical Imaging Group at the Department of Applied Physics, Kuopio, Finland). A medium filter was applied when the Kubios HRV software required smoothing abnormal peaks. Then, the root mean square of successive differences in R‐R intervals (RMSSD, in ms), that reflects primarily parasympathetic nervous system activity, was determined as time‐domain indices. In the frequency domain, analysis included the high frequencies (HF, 0.15–0.4 Hz) and the low frequencies (LF, 0.04–0.15 Hz) measured for 5 min in the supine position and 5 min in the standing position (Schmitt, Regnard, et al. 2018), with only the last 4 min time window for each position used for data processing. In accordance with the recommendations of the European Society of Cardiology and the North American Society of Pacing Circulation (1996), the normalized spectral indices (or normalized unit) were defined by the developers of the Kubios HRV Standard software v3.0.0 2 as HFnu = HF/(LF + HF) and LFnu = LF/(LF + HF) (Biosignal Analysis and Medical Imaging Group at the Department of Applied Physics, Kuopio, Finland; [Caminal et al. 2018]). Baseline HRV indices for the normoxia period were established from the average of the last 2 days before the first day of hypoxia (Figure 1). All HRV measurements were taken each morning upon waking, after urination, and while in a fasting state.

Nocturnal HR and SpO_2_ per hour of sleep during the night (was recorded with a device CheckMe O_2_ + oximeter, Viatom Technology Co Ltd, Shenzhen, China) placed on the participant's right thumb. Mean SpO_2_ during sleep (from lying down to waking up) were considered for the analysis.

A maximal aerobic power test was performed on participants' personal bicycles with a Hammer Saris 2 ergometer (CycleOps, Madison, WI, USA). The test started with a 4 min warm‐up (100 W), then 30 W were incremented every 2 min until exhaustion. Gas exchange was constantly monitored during the effort with a MetaMax analyzer (Cortex, Leipzig, Germany). To measure gas exchange, MetaMax analyzer calibration was systematically performed before the start of the test with the MetaSoft software (Cortex, Leipzig, Germany). During the test, the software was used to analyze gas exchange and determine VO_2_max as the mean oxygen consumption values of the last 30 s of the test.

### 2.4 Psychological Questionnaires

Sleep disturbance was measured with the Groningen Sleep Quality Scale (GSQS; Jafarian et al. 2008), which is a scale that rates sleep disturbance the night before between 0 (no disturbance) and 14 points (poor sleep).

AMS was measured with the Lake Louise questionnaire (R. C. Roach et al. 2018), which requires respondents to rate five symptoms on a scale from 0 to 3 (i.e., headache, nausea/vomiting, fatigue, dizziness/light‐headedness, and AMS clinical functional score). The AMS score for an individual is the sum of the score for the five symptoms. If the total score is less than 3, the AMS is weak or null from 3 to 5 is mild and greater than 6 corresponds to severe AMS.

Daily TL was determined with the following formula from Foster (1998): TL (in a.u.) = RPE × duration, where RPE is the rating of perceived exertion (from 0 to 10 on the Borg CR10 scale, 1998), and duration is the training time (in min).

Stress and emotions were measured with the Tension and Effort Stress Inventory (TESI) of Sveback (1983). It consists of 20 individual response items. Two items require the athlete to estimate “the degree of pressure, stress, challenge, or demand” that they are exposed to with respect to (1) situational factors and (2) their own body (i.e., internal factors). Two items are concerned with the degree of effort invested by the respondent in trying to cope with the pressure from (1) external factors and (2) internal factors associated with their own body. Sixteen items concern a list of 16 different emotions (8 pleasant emotions and 8 unpleasant emotions). The individual responds by selecting the appropriate level on a 1–7 scale ranging from “not at all” to “very much”. This standard version of the TESI was modified by the addition the five scales from the Telic State Measure (TSM) of Svebak and Murgatroyd (1985), which assesses (1) serious‐playful; (2) preferred planned‐preferred, (3) low felt arousal‐high felt arousal; (4) preferred low arousal‐preferred high arousal; and (5) low effort‐high effort. They were recorded using 7‐point scales consistent with the TESI. This type of modification has been used in previously published research (e.g., Males and Kerr 1996). The TSM is an instrument used to assess activation and relaxation states in individuals. Specifically, the TSM measures aspects of temporal orientation, distinguishing between two main states:

◦ Telic state: associated with a temporal orientation toward specific goals or activities. Individuals in this state are typically focused on tasks to be accomplished and may experience a high level of activation or stress related to pursuing these goals.
◦ Paratelic state: conversely, this state is characterized by a more relaxed and nondirective temporal orientation. Individuals in this state may feel more open to the present experience, less concerned with achieving specific goals, and thus more relaxed.

The TSM quantifies the relative prevalence of tension and relaxation states in an individual at a given time.

The Sports Grit Scale (SGS; Fruchart and Rulence‐Pâques 2024) is a tool that measures a personality trait combining perseverance and passion for long‐term goals in a specific sports domain. The SGS is composed of four dimensions, namely, “Achieving objectives” (4 items), “Giving up” (3 items), “Surpassing oneself” (3 items), and “Focusing on sport” (3 items). For all 13 items, responses are rated on a 5‐point Likert scale ranging from 1 (not at all like me) to 5 (very much like me).

### 2.5 Data and Statistical Analysis

For each period (normoxia, early, and middle acclimatization), data are reported as mean ± standard deviation (SD). Normality was checked using the Shapiro–Wilk test, and the assumption of data sphericity was assessed and corrected with the appropriate Greenhouse–Geisser or Huynh–Feldt correction. One‐way analysis of variance (ANOVA) with repeated measures (mean values of 3 periods: normoxia, early acclimatization, and middle acclimatization) and Fisher’s *post hoc* test were used to determine possible significant changes in sleep disturbance, sleep architecture, HRV, nocturnal HR, SpO_2_, TL, AMS, stress, and personality traits. For RMSSD indices, the values were log‐transformed to enable parametric statistical comparison. Then, the logarithm of RMSSD (LnRMSSD), which expresses the value on a 100‐point scale by multiplying by 20 (i.e., LnRMSSD × 20; Flatt et al. 2017) and the coefficient of variation of LnRMSSD (LnRMSSD_cv_), were determined to measure daily perturbations. Cohen's *d* effect size analysis (Cohen 1988) with 95% confidence interval (95% CI) was calculated to determine the magnitude of the effects between the 3 periods. A *d* < 0.20 was classified as trivial, a *d* ranging from 0.20 to 0.49 was classified as small, from 0.50 to 0.79 was classified as moderate, and a *d* > 0.80 was classified as large. Statistical analysis was performed using SigmaStat (Jandel Corporation, San Rafael, CA, USA), and *d* was calculated using a freely available spreadsheet (https://www.cem.org/effect‐size‐calculator). Statistical significance was set at *p* < 0.05.

## 3 Results

### 3.1 Sleep Parameters

No significant difference was observed across periods in nocturnal HR, total sleep time, sleep efficiency, latency, waking, NREM3, or REM (Table 2). However, a period effect was observed, with a significant increase for NREM1 (*p* < 0.007; *d* = 1.22; and 95% CI = 0.22–2.12) and NREM2 (*p* < 0.008; *d* = 0.67; and 95% CI = 0.26–1.54) during the early acclimatization period compared to the normoxic period and between early and middle acclimatization periods (*d* = 1.04 and 95% CI = 0.07 to 1.93 and *d* = 0.83 and 95%CI = 0.11 to 1.71, respectively). A significant decrease in SpO_2_ (*p* < 0.001) was found between normoxia and early (*d* = −3.08 and 95%CI = −1.68 to −4.22) and middle acclimatization (*d* = −3.36 and 95%CI = −1.68 to −4.22). In addition, a period effect was also found for sleep disturbance, with a significant increase for the GSQS score (*p* < 0.001, *d* = 1.24, and 95% CI = 0.24–2.14) between the normoxic and early acclimatization periods.

**TABLE 2:** Mean values (*M*) and standard deviation (SD) of sleep disturbance (GSQS), nocturnal heart rate (in bpm), % peripheral saturation in O_2_ (% SpO_2_), total sleep time (min), sleep efficiency (%), sleep onset latency (min), wakefulness after sleep onset (WASO; min), and sleep architecture (NREM1, NREM2, NREM3, and REM) recorded in the different periods.

		Normoxia		Hypoxia early acclimatization		Hypoxia middle acclimatization		ANOVA *F* and *p* values (period effect)		*d*
		*M*	SD	*M*	SD	*M*	SD	*F*	*p*	
	Sleep disturbance/11	2.5	1.1	4.9***	2.5	3.7	1.1	5.32	0.001	N versus EA = 1.24; N versus MA = 1.09; and MA versus EA = 0.62
Sleep parameters	Nocturnal HR (bpm)	56.9	9.6	61.9	5.5	60.1	7.0	1.62	0.22	N versus EA = 0.64; N versus MA = 0.38; and MA versus EA = 0.29
	% SpO_2_	96.4	1.4	90.9***	2.1	91.7***	1.4	66.52	0.001	N versus EA = −3.08; N versus MA = −3.36; and MA versus EA = −0.45
	Total sleep time (min)	495.9	16.3	506.8	18.2	478.0	14.8	0.28	0.75	N versus EA = 0.63; N versus MA = −1.15; and MA versus EA = 1.74
	Sleep efficiency (%)	90.7	4.5	91.6	3.3	91.1	2.9	1.09	0.35	N versus EA = 0.23; N versus MA = 0.11; and MA versus EA = 0.16
	Sleep onset latency (min)	28.2	15.9	28.2	16.5	30.4	15.0	0.16	0.84	N versus EA = 0.01; N versus MA = 0.14; and MA versus EA = −0.13
	WASO (min)	52.2	19.3	47.4	21.0	47.7	16.9	0.39	0.68	N versus EA = −0.24; N versus MA = −0.25; and MA versus EA = −0.02
	NREM1 (min)	21.8	3.1	25.9*	3.6	21.6	4.6	6.42	0.007	N versus EA = 1.22; N versus MA = −0.05; and MA versus EA = 1.04
	NREM2 (min)	201.2	55.9	238.5*	55.8	195.6	47.1	6.12	0.008	N versus EA = 0.67; N versus MA = −0.11; and MA versus EA = 0.83
	NREM3 (min)	104.3	12.3	98.0	22.7	109.1	29.06	1.94	0.16	N versus EA = −0.35; N versus MA = 0.22; and MA versus EA = −0.43
	REM (min)	172.0	69.1	148.3	32.6	170.0	63.9	0.27	0.76	N versus EA = −0.44; N versus MA = −0.03; and MA versus EA = −0.43

*Note: F* and *p* values correspond to the ANOVA with repeated measures.

*Abbreviations:* EA = hypoxia early acclimatization, MA = hypoxia middle acclimatization, and N = normoxia.

*p < 0.05 compared to normoxia and hypoxia middle acclimatization and ***p < 0.001 compared to normoxia.

### 3.2 Heart Rate Variability

In the supine position, there was no significant difference in the RMSSD, LnRMSSD, LFnu, HFnu, or LFnu/HFnu ratio (Table 3). However, a period effect was observed, with a significant increase (*p* < 0.003, *d* = 1.76, and 95%CI = 0.66–2.70) in LnRMSSD_cv_ between the normoxic and the middle acclimatization period. In the standing position, we observed a significant decrease (*p* < 0.001) in RMSSD and LnRMSSD between normoxia and both early acclimatization periods (*d* = −1.60 and 95% CI = −0.54 to −2.53 and *d* = −1.59 and 95% CI = −0.53 to −2.52, respectively) and middle acclimatization periods (*d* = −1.49 and 95% CI = −0.45 to −2.41 and *d* = −1.40 and 95% CI = −0.37 to −2.31, respectively). Compared to normoxia, a significant increase (*p* < 0.005) in LFnu was found during the early and middle acclimatization periods (*d* = 1.07 and 95% CI = 0.09 to 1.95 and *d* = 1.10 and 95% CI = 0.12 to 1.99, respectively) with a significant decrease (*p* < 0.005) in HFnu during the early and middle acclimatization periods (*d* = −1.07 and 95% CI = −0.09 to −1.95) and (*d* = −1.03 and 95% CI = −0.05 to −1.91, respectively). However, no significant changes were observed for the LnRMSSD_cv_ or LFnu/HFnu ratio.

**TABLE 3:** Mean values (*M*) and standard deviation (SD) of heart rate variability (RMSSD, LnRMSSD, LnRMSSDcv, LFnu, HFnu, and Ratio LF nu/HFnu).

			Normoxia		Hypoxia early acclimatization		Hypoxia middle acclimatization		ANOVA F and *p* values (period effect)		*d*
			*M*	SD	*M*	SD	*M*	SD	*F*	*p*	
Heart rate variability	Supine	RMSSD (ms)	73.7	23.3	71.8	24.3	73.7	26.1	0.08	0.91	N versus EA = −0.08; N versus MA = 0.0; and MA versus EA = −0.08
		LnRMSSD (ms)	84.9	6.68	84.4	7.04	84.7	7.9	0.06	0.94	N versus EA = −0.07; N versus MA = −0.03; and MA versus EA = −0.04
		LnRMSSDcv (%)	3.55	2.26	5.42	3.18	7.65**	2.40	8.10	0.003	N versus EA = 0.68; N versus MA = 1.76; and MA versus EA = −0.79
		LFnu	0.49	0.20	0.56	0.17	0.49	0.08	2.07	0.16	N versus EA = 0.38; N versus MA = 0.0; and MA versus EA = 0.53
		HFnu	0.51	0.21	0.43	0.12	0.51	0.08	2.07	0.16	N versus EA = −0.47; N versus MA = 0.0; and MA versus EA = −0.78
		Ratio LFnu/HFnu	1.36	1.14	1.45	0.61	1.03	0.29	1.24	0.32	N versus EA = 0.10; N versus MA = −0.40; and MA versus EA = 0.88
	Standing	RMSSD (ms)	57.70	29.11	24.30***	4.81	26.60***	4.41	12.77	0.001	N versus EA = −1.60; N versus MA = −1.49; and MA versus EA = −0.50
		LnRMSSD (ms)	33.93	5.38	27.59***	1.68	28.39***	1.5	12.69	0.001	N versus EA = −1.59; N versus MA = −1.40; and MA versus EA = −0.50
		LnRMSSDcv(%)	0.38	0.2	0.40	0.3	0.29	0.2	0.35	0.70	N versus EA = 0.08; N versus MA = −0.45; and MA versus EA = 0.43
		LFnu	0.63	0.15	0.79*	0.15	0.78*	0.12	7.59	0.005	N versus EA = 1.07; N versus MA = 1.10; and MA versus EA = 0.07
		HFnu	0.36	0.15	0.20*	0.15	0.22*	0.12	7.59	0.005	N versus EA = −1.07; N versus MA = −1.03; and MA versus EA = −0.15
		Ratio LFnu/HFnu	3.71	6.02	6.24	4.2	4.98	3.15	1.14	0.33	N versus EA = 0.49; N versus MA = 0.26; and MA versus EA = 0.34

*Note: F* and *p* values correspond to the ANOVA with repeated measures.

Abbreviations: EA = hypoxia early acclimatization, MA = hypoxia middle acclimatization, and N = normoxia.

**p < 0.002 compared to normoxia, ***p < 0.001 compared to normoxia, and *p < 0.05 compared to normoxia.

### 3.3 Psychological Stress

**FIGURE 2:**
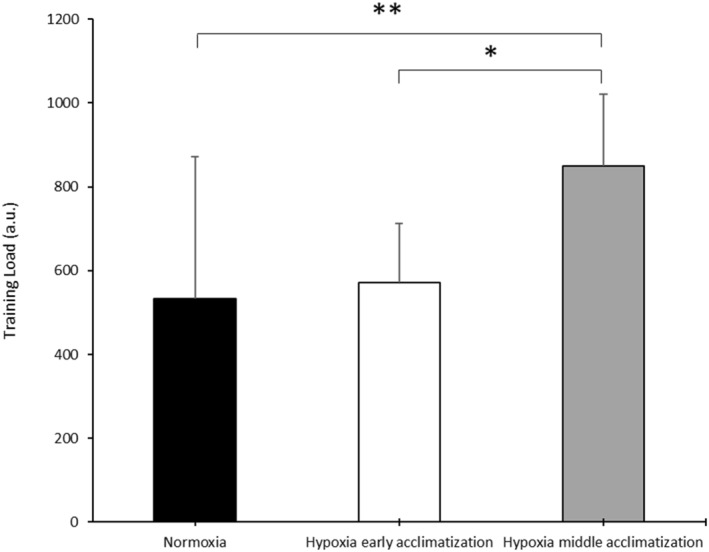
Mean and standard deviation (±SD) of the training load recorded in different periods. **p < 0.005 compared to hypoxia middle acclimatization and *p < 0.05 compared to hypoxia middle acclimatization.

Concerning the TESI (Table 4), a significant effect was observed between the normoxic and middle acclimatization periods with an increase (*p* < 0.05) in external tension (*d* = 1.21 and 95% CI = 0.21–2.11). However, no significant change was found in the AMS score. A significant increase (*p* < 0.005) was observed in TL (Figure 2) between normoxia and middle acclimatization and between early acclimatization and middle acclimatization (*d* = 1.19 and 95% CI = 0.19–2.08) and (*d* = 1.78 and 95% CI = 0.68 to 2.72, respectively).

**TABLE 4:** Mean (*M*) and standard deviation (SD) of stress, emotions/arousal, and acute mountain sickness recorded in different periods.

		Normoxia		Hypoxia early acclimatization		Hypoxia middle acclimatization		ANOVA F and *p* values (period effect)		*d*
		*M*	SD	*M*	SD	*M*	SD	*F*	*p*	
TESI	External tension/7	1.25	0.4	2.32	1.3	2.83*	1.8	4.15	0.03	N versus EA = 1.11; N versus MA = 1.21; and MA versus EA = −0.32
	Effort external/7	1.75	1.5	2.31	1.2	3.1	2.1	1.66	0.22	N versus EA = 0.41; N versus MA = 0.74; and MA versus EA = −0.46
	Δ tension/effort external	0.50	1.3	0.00	1.1	0.36	0.46	0.57	0.58	N versus EA = −0.42; N versus MA = −0.14; and MA versus EA = −0.43
	Tension internal/7	3.25	1.9	3.31	1.3	3.3	1.7	0.01	0.98	N versus EA = 0.04; N versus MA = 0.03; and MA versus EA = 0.01
	Effort internal tension/7	3.25	2.0	3.44	1.5	3.37	1.9	0.04	0.95	N versus EA = 0.11; N versus MA = 0.06; and MA versus EA = 0.04
	Δ tension/effort internal	0.00	0.9	0.13	0.3	0.03	0.6	0.10	0.89	N versus EA = 0.19; N versus MA = 0.04; and MA versus EA = 0.21
	Pleasant emotions/56	20.93	7.8	19.5	6.7	20.0	6.2	0.85	0.44	N versus EA = −0.20; N versus MA = −0.13; and MA versus EA = −0.08
	Unpleasant emotions/56	12.0	3.1	12.5	6.6	12.4	4.2	0.10	0.90	N versus EA = 0.1; N versus MA = 0.11; and MA versus EA = 0.02
	Δ pleasant/unpleasant emotions/48	8.9	9.1	7.1	9.2	8.2	6.5	0.39	0.67	N versus EA = −0.20; N versus MA = −0.09; and MA versus EA = −0.14
Acute mountain sickness	AMS/12	0.6	0.7	0.8	0.6	0.6	0.6	2.00	0.11	N versus EA = 0.31; N versus MA = 0.0; and MA versus EA = 0.33

*Note: F* and *p* values correspond to the ANOVA with repeated measures.

Abbreviations: EA = hypoxia early acclimatization, MA = hypoxia middle acclimatization, and N = normoxia.

*p < 0.05 compared to normoxia.

**FIGURE 3:**
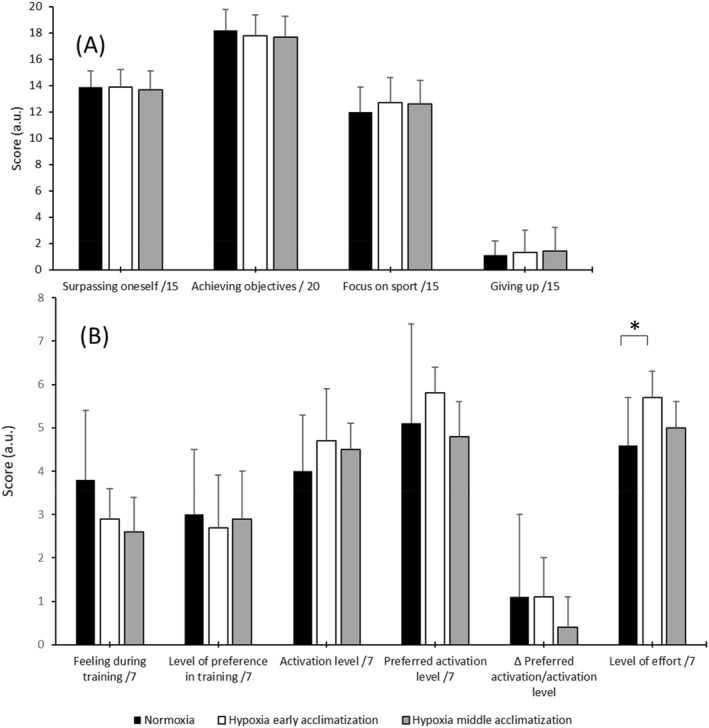
Mean and standard deviation of sport grit scale (A) (scores are on 15 for surpassing oneself, focus on sport, and giving up and on 20 for achieving objectives) and telic state measure (B) (scores are on 7) recorded in different periods. *p < 0.05 compared to hypoxia early acclimatization.

Concerning the SGS (Figure 3A), no significant difference was found between the different periods. Nevertheless, in the TSM (Figure 3B), a significant increase (*p* < 0.05) in the level of effort was observed between the normoxic and the early acclimatization periods (*d* = 1.24 and 95% CI = 0.24–2.14).

## 4 Discussion

The aim of this exploratory study was to investigate the effects of LHTLH on sleep, HRV, and psychological stress in 10 elite/international level female track endurance cyclists. The main findings indicate that sleep disturbance, perceived effort, and perturbation of sleep architecture significantly increased during the LHTLH early acclimatization period but return to normal sleep architecture and quality in the middle acclimatization period. This result suggests that an acclimatization period of 4 days is required to recover restorative sleep, whereas HRV remains altered, with an increase in external tension persisting for at least 10 days.

### 4.1 Sleep Parameters

The most important finding of this study is the significant change in sleep architecture and sleep disturbance observed by the participants during the LHTLH early acclimatization period before returning to normal during the LHTLH middle acclimatization period. The disruption of sleep architecture during the early acclimatization period has been previously documented by Mizuno et al. (2005), who observed an increase in time spent in NREM1—a phase of light nonrestorative sleep—during the first four nights of a 6‐day exposure at 3776 m. This aligns also with previous findings reporting that hypoxic exposure disrupts sleep, impairing physical recovery, cognitive performance, and metabolic efficiency, all crucial for elite‐level athletes (Weil 2004). Although the LHTLH method offers athletes significant physiological benefits (Brocherie et al. 2018) with hypoxia‐inducible factor‐1α subunit, vascular endothelial growth factor, myoglobin, peroxisome proliferator‐activated receptor‐gamma coactivator 1‐α, and mitochondrial transcription factor AmRNA levels increase that boost performance; however, the present results demonstrate that, compared to sea‐level training, the LHTLH early acclimatization period disrupted sleep, which could potentially lead to overreaching. Careful planning and monitoring during LHTLH intervention appears as a prerequisite to effectively maximize the physiological adaptations and performance gains while minimizing drawback.

In contrast, Hoshikawa et al. (2007) reported that acute exposure to normobaric hypoxia equivalent to 2000 m altitude resulted in a reduction of slow‐wave sleep (NREM3) in athletes. Similarly, Heinzer et al. (2016) found no significant increase in NREM1 or NREM2 but observed a significant decrease in NREM3 and REM sleep during acute (one night) normobaric hypoxia at an equivalent altitude of 3450 m. More recently, Hrozanova et al. (2021) reported that in elite endurance athletes, light sleep decreased whereas deep sleep increased during a 2‐week training camp at 1800 m. These discrepancies may be attributed to differences in altitude across studies and warrant further investigation.

During LHTLH, an *increase in light sleep (NREM1)* whereas other sleep stages (NREM2–3 and REM) remain stable could have several potential consequences, with perception of poor sleep. Indeed, it has been reported (Staner 2003) that light sleep is less restorative compared to deeper sleep stages, even if the total sleep duration remains adequate. The author suggests that increased time in light sleep might indicate more frequent awakenings or an inability to progress smoothly through deeper sleep stages, leading to sleep fragmentation. There are also potential psychophysiological consequences in perceived fatigue and sleepiness with cognitive impairment with difficulties in focus and decision‐making during the day and impacts on autonomic nervous system imbalance by affecting HRV.

The lack of significant alteration in NREM3 during acute hypoxia likely could reflect the brain's prioritization of restorative processes under stress. This adaptive response could ensure the preservation of critical physiological benefits, even in challenging conditions such as hypoxia. It has been reported by Weil (2004) that hypoxia‐induced fatigue can elevate homeostatic sleep pressure, making it harder to disrupt NREM3 even in the presence of physiological stress.

In parallel to the deterioration of sleep architecture, we observed an increase in sleep disturbances during the LHTLH early acclimatization period. However, AMS score remained low, confirming that LHTHL intervention between 2500 m (F_i_O_2_ = 15.09%) and 3000 m (F_i_O_2_ = 14.68%) of simulated altitude did not induce headaches, gastrointestinal symptoms, dyspnea, or dizziness. This finding has been also reported previously by Brugniaux et al. (2006) in athletes during “living high‐training low” in hypoxic rooms with an O_2_ fraction corresponding to 2500–3500 m.

Another relevant finding in the present study is the reversal effect of the LHTLH middle acclimatization period with a lack of significant changes in sleep architecture or sleep disturbance observed in reference to the normoxic period. This result suggests that an acclimatization period of 4 days is necessary before returning to normal sleep architecture and quality.

It is also noteworthy that, with an average total sleep duration of more than 8 h per night and a sleep efficiency consistently above 85%, the participants appear to have a quantity and quality of sleep that align with the recommendations for elite athletes (Roberts et al. 2018). However, Walsh et al. (2020) showed that most elite athletes sleep less than 7 h per night, often with fragmented sleep caused by stress and anxiety. The fact that the experimental protocol took place during a preseason‐training period, with no competitions scheduled for several weeks, could explain the good sleep quality and quantity observed in the participants.

### 4.2 Heart Rate Variability

We studied the standing/supine responses of HRV to orthostatic stress, as it reflects the adaptation of the sinus node. HRV imbalance was observed in both LHTLH early and middle acclimatization periods compared to the normoxic period, with a decrease in vagal activity (as shown by the decreases in HFnu, RMSSD, and LnRMSSD values in the standing position). Similar findings have been previously reported in acute simulated hypoxia corresponding to 4000 m (Guger et al. 2008). Moreover, in our study, LF_nu_ increased during the early and middle acclimatization periods in comparison with normoxia. Because LF_nu_ bands reflects both the sympathetic and parasympathetic influence (Schmitt, Regnard, et al. 2018; Botek et al. 2015), the results of the present study confirm a HRV imbalance during the LHTLH intervention. Moreover, the significant decrease in HF_nu_ observed in the standing position in both periods of hypoxia seems to explain a high level of psychophysiological stress (Dimitriev and Saperova 2015), which may be linked to the increase of effort and external tension and recorded in the LHTLH early and middle acclimatization periods, respectively, but also to the increase in the TL observed in the LHTLH middle acclimatization period. These results appear of practical relevance in sporting context where using HRV metrics could be helpful to tailor TL and chronic altitude‐related acclimatization strategies, ensuring optimal recovery and reducing overreaching risks during such interventions.

### 4.3 Psychological Stress

Although Kerr and Kuk (2001) reported that exercise intensity did not influence external tension during aerobic exercise, the significant increase in external tension found during the LHTLH middle acclimatization period seems to indicate the importance of hypoxic stress on the psychological state of the athletes. Indeed, this finding suggests that LHTLH induces an exacerbation of stress. The significant increase in the level of effort observed during the early acclimatization period seems to confirm the deleterious impact of altitude/hypoxic exposure on the psychological responses of athletes as previously reported by Jeffries et al. (2019). However, contrary to Shukitt‐Hale and Lieberman (1996) who reported a negative effect on mood during the two first days of altitude exposure at 4300 m, the results of the present study revealed that LHTLH did not significantly alter emotions during both early and middle acclimatization periods. This contradictory finding may be due to the difference in hypoxic stress between both studies. Moreover, the lack of significant changes observed in the perseverance of effort and the consistency of interest, as evaluated by the SGS, suggests that these personality traits remain stable despite the decrease in physiological response (i.e., SpO_2_ and HRV) and sleep disturbances and the increase in effort and external tension.

### 4.4 Limitations

This study has several limitations, including the small sample size and the lack of control group performing the same training program in normoxia. In addition, findings should be interpreted with caution, as this experiment was conducted in normobaric hypoxia that is known for inducing slight physiological differences when compared to hypobaric hypoxia (Millet and Debevec 2020). Specifically, the ventilatory acclimatization may be poorly translated from one condition to the other one (Fulco et al. 2013). Furthermore, the protocol implemented in this study does not allow for a precise determination of the potential impact of the increased TL during the LHTLH middle acclimatization period on HRV and external tension. Lastly, the women's perception of variations in performance during the menstrual cycle could cause significant changes in psychological state (Carmichael et al. 2021) as well as perturbations of HRV (Schmalenberger et al. 2020) and possibly also fluctuations in sports performance (Elliott‐Sale et al. 2021), which were not possible to consider in this study. Further investigations are warranted in a large sample of women in the same menstrual cycle period to determine its potential influence on sleep, HRV, and psychological stress during training in elite‐level female athletes during LHTLH.

## 5 Conclusion

This study showed that, in the experimental conditions tested here, the “Living High‐Training Low and High” early acclimatization period induced significant changes caused by an increase in psychological stress with perceived effort, sleep disturbance, perturbation of sleep architecture, and heart rate variability. However, measurements in the middle acclimatization phase indicate that the athletes recovered restorative sleep and decreased psychological stress, enabling them to increase their training loads. However, the cardiac autonomic responses remain altered with an increase of external tension for at least 10 days.

### 5.1 Perspectives

Our findings add new perspectives on the effects of “Living High‐Training Low and High” by confirming the importance of the acclimatization period during the chronic altitude/hypoxic training camp. Coaches and athletes who use “Living High‐Training Low and High” approach in their periodization, might ensure sufficient recovery in the early stages of acclimatization while gradually increasing training loads in subsequent days to maximize performance gains.

## Author Contributions

**Thibaud Pirlot:** data curation, investigation, writing – original draft. **Thibaud Mihailovic:** data curation, investigation. **Philippe Gimenez:** data curation, investigation, funding acquisition. **Gregoire P. Millet:** writing – review, methodology. **Franck Brocherie:** writing – review, methodology. **Eric Fruchart:** writing – review, methodology. **Gilles Ravier:** writing – review, methodology. **Bertrand Baron:** methodology. **Romain Bouzigon:** resources, methodology. **Sandrin Guirronet:** resources. **Emmanuel Brunet:** resources. **Alain Groslambert:** data curation, investigation, writing – original draft, funding acquisition.

## Conflicts of Interest

The authors declare that there are no conflicts of interest that could be perceived as prejudicing the impartiality of the research report.
